# A Novel Berbamine Derivative Inhibits Cell Viability and Induces Apoptosis in Cancer Stem-Like Cells of Human Glioblastoma, via Up-Regulation of miRNA-4284 and JNK/AP-1 Signaling

**DOI:** 10.1371/journal.pone.0094443

**Published:** 2014-04-14

**Authors:** Fan Yang, Sangkil Nam, Christine E. Brown, Robin Zhao, Renate Starr, David A. Horne, Linda H. Malkas, Richard Jove, Robert J. Hickey

**Affiliations:** 1 Translational Biomarker Discovery Core, Beckman Research Institute, City of Hope Comprehensive Cancer Center, Duarte, California, United States of America; 2 Molecular Medicine, Beckman Research Institute, City of Hope Comprehensive Cancer Center, Duarte, California, United States of America; 3 Cancer Immunotherapeutics & Tumor Immunology, Beckman Research Institute, City of Hope Comprehensive Cancer Center, Duarte, California, United States of America; 4 Molecular and Cellular Biology, Beckman Research Institute, City of Hope Comprehensive Cancer Center, Duarte, California, United States of America; 5 Molecular Pharmacology, Beckman Research Institute, City of Hope Comprehensive Cancer Center, Duarte, California, United States of America; 6 Vaccine & Gene Therapy Institute of Florida, Port St. Lucie, Florida, United States of America; University of Florida, United States of America

## Abstract

Glioblastoma (GBM) is the most common primary brain tumor, accounting for approximately 40% of all central nervous system malignancies. Despite standard treatment consisting of surgical resection, radiotherapy and/or chemotherapy, the prognosis for GBM is poor; with a median survival of 14.6 months. The cancer stem cell or cancer-initiating cell model has provided a new paradigm for understanding development and recurrence of GBM following treatment. Berbamine (BBM) is a natural compound derived from the *Berberis amurensis* plant, and along with its derivatives, has been shown to exhibit antitumor activity in several cancers. Here, we reported that a novel synthetic Berbamine derivative, BBMD3, inhibits cell viability and induces apoptosis of cancer stem-like cells (CSCs) in a time- and dose-dependent manner when the CSCs from four GBM patients (PBT003, PBT008, PBT022, and PBT030) were cultured. These CSCs grew in neurospheres and expressed CD133 and nestin as markers. Treatment with BBMD3 destroyed the neurosphere morphology, and led to the induction of apoptosis in the CSCs. Induction of apoptosis in these CSCs is dependent upon activation of caspase-3 and cleavage of poly (ADP-ribose) polymerase (PARP). MicroRNA-4284 (miR-4284) was shown to be over-expressed about 4-fold in the CSCs following BBMD3 treatment. Furthermore, transfection of synthetic anti-sense oligonucleotide against human miR-4284 partially blocked the anticancer effects of BBMD3 on the GBM derived CSCs. BBMD3 also increased phosphorylation of the c-Jun N-terminal kinase (JNK)/stress-activated protein kinase (SAPK), resulting in an increase expression of phosphorylated c-Jun and total c-Fos; the major components of transcriptional factor AP-1. The JNK-c-Jun/AP-1 signaling pathway plays an important role in the induction of apoptosis in response to UV irradiation and some drug treatments. Targeting glioblastoma stem-like cells with BBMD3 is therefore novel, and may have promise as an effective therapeutic strategy for treating GBM patients.

## Introduction

Glioblastoma (GBM) is the most common and lethal primary brain tumor. Despite current advances in multimodality therapy, which include surgery, radiotherapy and chemotherapy, prognosis remains very poor for patients, who typically have a median survival time of less than 15 months [1], [2]. The majority of GBM lesions rapidly develop from a less malignant precursor lesion for which there is little or no clinical, radiological, or morphologic evidence, and it has been demonstrated that a highly tumorigenic subpopulation of cancer cells, called GBM stem cells, promotes resistance to chemo- and radio- therapy [3]–[5]. These cancer stem cells or tumor-initiating cells share some critical characteristics with normal neural stem cells, including expression of several biomarkers, and the ability for self-renewal, differentiation and proliferation. Due to the poor prognosis for GBM patients following currently available therapies, development of more effective protocols for treating GBM is urgently needed. However progress slowing protocol development remains dependent upon further enhancement of our understanding of the processes driving cancer invasion, the onset of resistance to therapeutic interventions and mechanisms driving tumor recurrence in GBM patients. Thus, the effective treatment of GBM requires directly targeting these GBM stem cells within the tumor mass, since they are the cells that are resistant to standard therapies [6]. In this regard, Brown et al [7] recently provided a rationale for developing an immunotherapeutic approach for eradicating the GBM stem cell population by reporting that human tumor stem/initiating cells from GBM patients could be recognized and killed by CD8^+^ cytotoxic T lymphocytes.

In addition to this immunological approach, microRNA (miRNA), which is a relatively new class of small non-coding RNA molecule found in eukaryotic cells, has been shown to regulate a wide spectrum of gene expression patterns via a post-transcriptional mechanism [8]. And a considerable body of evidence now indicates that miRNAs play key roles in the pathogenesis of cancer, and can function either as oncogenes or tumor suppressors [9]. It has also been reported that high expression of miR-196 and miR10b in GBM patients correlates with a poor prognosis [10], and that down-regulation of miR-128 leads to reduction in the self-renewal ability of glioma stem cells by inhibiting Bmi1 gene expression. Thus, miRNAs are rapidly emerging as promising targets for the development of novel but highly selective anticancer therapeutic agents.

Several years ago, Berbamine (BBM), a natural bis-benzylisoquinoline alkaloid, was identified from the traditional Chinese medicine *Berberis amurensis*, and along with several of its derivatives, was reported to have potent antitumor activity toward lymphoma, myeloma, hepatocellular carcinoma, lung cancer and breast cancer and low non-specific toxicity [12]–[16]. BBM was reported to induce apoptosis in human myeloma, and to suppress growth, migration and invasion of highly-metastatic human breast cancer cells through inhibiting NF-kappaB (NF-KB) activity and its downstream targets, such as cyclin D1, Bcl-xL and survivin [13], [14]. Thus the low non-specific toxicity of BBM, and some of its derivatives, makes continued development of these novel compounds potentially promising as effective anti-cancer drugs for a variety of types of cancer. Toward this goal, our lab examined thirteen novel BBM derivatives (BBMDs), which were synthesized from natural BBM. We found that BBMD3 is the most potent of this series of novel BBMDs for killing cancer cells. BBMD3 exhibited over a 6-fold increase in anticancer activity toward melanoma and prostate cancer cells as compared to natural BBM. This was attributed to inhibition of the JAK2/STAT3 signaling pathway [17]. Recently, we also reported that BBMD3 inhibits cell viability, and induces apoptosis in human osteosarcoma cells, which, in this case, was attributed to activation of the JNK/AP-1 signaling pathway [18].

The superfamily of mitogen-activated protein kinases (MAPKs) includes: c-Jun N-terminal protein kinase (JNK)/stress-activated protein kinase (SAPK); p38; and extracellular signal-regulated kinase (ERK). In general, JNK and p38 are key mediators of the response to stress and inflammation, while the ERK cascade is most often induced in response to growth factor stimulation [19], [20]. The JNK stress pathway participates in many different intracellular processes, including cell growth, differentiation, transformation and apoptosis [21], [22]. The JNK protein kinases are encoded by three genes, of which *Jnk1* and *Jnk2* are expressed by all tissues, and the *Jnk3* gene is confined to a more limited pattern of expression such as in brain and heart [22]. JNK was originally identified by its ability to specifically phosphorylate the transcription factor c-Jun on its N-terminal trans-activation domain at Ser63 and Ser73 [23]. c-Jun is a major component of activating protein-1 (AP-1), which is a dimeric transcriptional factor, and is composed of proteins from several gene families [24]. Though the JNK/c-Jun or JNK/AP-1 pathway have dual roles in apoptosis, it is clear that activation of the JNK pathway is involved in the induction of apoptosis by specific cell death stimuli, such as UV irradiation and treatment with certain drugs [25]–[27].

In our present study, we show that a novel synthetic BBM derivative (BBMD3) induces apoptosis in cancer stem-like cells, which are cultured from GBM patients. We also show that cell killing is associated with up-regulation of miRNA-4284 and the JNK/AP-1 signaling pathway.

## Materials and Methods

### Reagents and Antibodies

BBMD3 was synthesized by reacting natural BBM with NH_2_-containing substitutents. The structure and purity of the products were analyzed using HNMR spectroscopy. BBMD3 displayed over 98% purity by NMR analysis. Human miRNA inhibitors (synthetic oligonucleotides) against hsa-miRNA-4284 and has-miRNA-27a were purchased from GeneCopoeia. RNAiFect Transfection reagent was purchased from Qiagen Inc. Recombinant human epidermal growth factor (EGF) and fibroblast growth factor basic (FGF) were obtained from R&D Systems Inc., and Accutase was purchased from Innovative Cell Technologies. B-27 serum-free supplement (50X) for growth and maintenance of neurons was obtained from Gibco. Heparin (1000 units/ml) was purchased from APP Pharmaceuticals, LLC. Horseradish peroxidase-labeled anti-mouse and anti-rabbit secondary antibodies were purchased from GE Healthcare. Anti-nestin antibody was purchased from EMD Millipore. All other antibodies were purchased from Cell Signaling, Inc.

### Cell Cultures

GBM specimens used in this study, (were evaluated by an attending neuropathologist, and assigned a grade IV classification using the World Health Organization (WHO) established guidelines), were obtained from patients undergoing surgical treatment at the City of Hope Medical Center, in accordance with Institutional Review Board (IRB)-approved protocols. Tumor specimens were given unique patient brain tumor (PBT) numbers, (i.e. PBT003, PBT008, PBT022 and PBT030). GBM neurosphere (NS) cultures were cultured and expanded as previously described [7]. Briefly, NS cultures were maintained in neural stem cell media (DMEM/F-12), supplemented with 1∶50 B27 serum-free supplement, 20 ng/mL EGF, 20 ng/ml FGF basic and 0.84 unit/ml of heparin. Normal human neurospheres or non-malignant neurospheres were cultured from human fetal brain tissue under the same culture condition as the GBM neurospheres. The established human glioblastoma cell line U87, was purchased from the American Type Culture Collection (ATCC), and both the U87 and the attached or differentiated cells derived from the GBM neurospheres (stem-like cells), were cultured in DMEM media (containing L-glutamine), supplemented with 10% fetal bovine serum (FBS) and 1% Antibiotic-Antimycotic (AA). All cultured cells were grown at 37°C in a humidified atmosphere containing 5% CO_2_.

### Treatment of BBMD3

BBMD3 was dissolved in dimethyl sulfoxide (DMSO) and diluted with cell culture medium. To obtain single cells from GBM neurospheres, neurospheres were treated with accutase for 10 min at 37°C and passed through a cell strainer (70 µm nylon). Cells were then seeded in cell culture flask or dishes with complete stem cell culture medium according to the protocol outlined in the experimental design section. Five hours later, different concentrations of diluted BBMD3 were added to the cells; while the controls received equal amount of only the vehicle.

### Cell Viability Assays

Cell viability assays were performed with the CellTiter 96 Aqueous One Solution Cell Proliferation Assay from Promega, which contains 3-(4,5-dimethylthiazol-2-yl)-5-(3-carboxymethoxyphenyl)-2-(4-sulfophenyl)-2H-tetrazolium (MTS). Each well of a 96-well plate was seeded with 5000 cells suspended in culture medium. Cells were treated with different concentrations of drug. After 24 h or 48 h of treatment, MTS was added to the cells according to the supplier’s protocol, and the absorbance of the reaction product formed by the cultured cells was measured at 490 nm using an ELISA plate reader.

### Apoptosis Assay

Cells (2×10^5^) derived from the neurospheres were seeded in 6-well plates with complete stem cell medium. The following day, the cells were treated for an additional 48 hours with the indicated concentrations of BBMD3. After treatment, all cells were collected, and the apoptotic cells were detected using an Annexin V-FITC Apoptosis Detection Kit (BD Biosciences). The cells were stained with Annexin V-FITC and propidium iodide (PI) according to the supplier’s instructions. Viable and apoptotic cells were detected by flow cytometry in our Analytical Cytometry Core here at the City of Hope National Medical Center. Apoptotic cells include both the early apoptotic portion (Annexin V-positive) and the late apoptotic portion (Annexin V and PI-positive).

### Photography

Normal NS culture, untreated GBM NS cultures derived from patient brain tumors (i.e., PBT003, PBT008, PBT022, PBT030) or PBT003 NS treated with different concentrations of BBMD3 were photographed at 10X magnification using a Nikon ECLIPSE TE2000-U microscope.

### Total Protein Preparation and Protein Assay

Following a 24 hour incubation with 5 µM BBMD3, cells derived from patient brain tumors (i.e., PBT003, PBT008, PBT022 and PBT030) were collected and washed with cold phosphate-buffered saline (PBS). Then, each cell pellet was added to 150 µl of Cell Signaling Lysis buffer (EMD Millipore), into which we had placed a protease inhibitor cocktail tablet (Roche, Inc., Indianapolis, IN). Following three successive cycles of freezing in a dry ice/ethanol bath and thawing in a 37°C water bath, the total protein concentration of the lysate was determined using a Bio-Rad Protein Assay kit.

### Immunoblotting Analysis

Twenty micrograms of total protein were resolved through a pre-cast 4–15% gradient Tris-HCl Polyacrylamide gel purchased from BIO-RAD (Hercules, CA). After gel electrophoresis, the proteins were transferred to Hybond-C membranes (Amersham), blocked for 1 hour at room temperature (RT) in 10% non-fat dry milk, which was dissolved in PBST (1X PBS containing 0.1% Tween-20), and incubated overnight at 4°C with primary antibodies (recognizing the proteins outlined in the text) suspended in PBST containing 2% non-fat dry milk. After washing the membranes in PBST, horseradish peroxidase labeled anti-mouse or anti-rabbit secondary antibodies were incubated with the membranes for 1 hour at RT. The location on the Hybond-C membrane of the antigenic proteins specifically bound to the antibodies was detected with SuperSignal West Pico substrate (Pierce).

### Quantitative Analysis of microRNAs

Total microRNA was isolated from PBT003, PBT008, PBT022 and PBT030 neurospheres following a 15 hour of treatment with 5 µM BBMD3 or the vehicle control using the miRNeasy Mini kit (Qiagen). Quantification of the relative abundance of each individual microRNA, with or without BBMD3 treatment, was completed in the Integrative Genomics Core at the City of Hope National Medical Center. Briefly, micro-RNA sequencing was performed using the Illumina HiSeq2000 following the manufacturer’s protocol (TruSeq Small RNA Sample Prep kit, Illumine, Inc.) with minor optimization. 500 ng of micro-RNA was ligated to the 3′ adapter (5′ TCTCTGTAGGCACCATCAATC) with T4 RNA ligase 2 for 1 hour at 22°C. The unligated 3′ adaptors were blocked by annealing them with RT primer (5′ ATTGATGGTGCCTACAGAGA) at 75°C for 5 min and 25°C for 15 minutes. The quantified denatured micro-RNA library was loaded in 1 ml of hybridization buffer to a final concentration of 10 pM.

### Treatment of micro-RNA Inhibitors

8000 single cells derived from PBT008 and PBT030 neurospheres in complete stem cell culture media were seeded into each well of 96-well plates. Five hours later, 60 nM miR-4284 or miR-27a anti-sense inhibitors were transfected into the cells by RNAiFect transfection reagent. The control cells were transfected with a control micro-RNA inhibitor. 24 hours following transfection with micro-RNA inhibitors, 5 µM BBMD3 was added to the cell culture plates. 24 hours following BBMD3 treatment, cell viability was determined.

### Statistics

Student’s *t* test was used to evaluate the statistical significance of differences between two groups and *p*<0.05 was considered statistically significant.

## Results

### Neurospheres Cultured from Specimens of GBM Patients Exhibit Characteristics of Cancer Stem-cells

**Figure 1:**
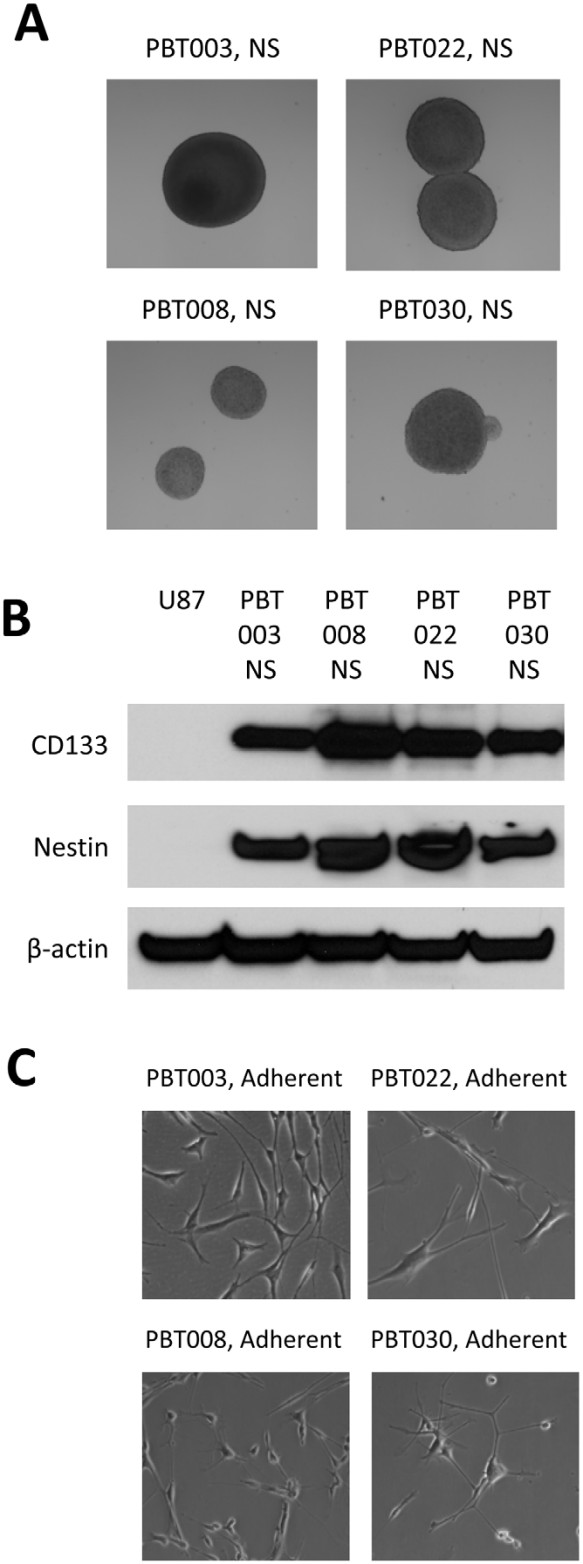
Neurospheres cultured from four GBM patients, PBT003, PBT008, PBT022 and PBT030 exhibited morphological characteristics of neural stem cells. (**A**) Morphology of PBT003, PBT008, PBT022 and PBT030 neurospheres in stem cell culture medium. (**B**) GBM neurospheres expressed specific biomarkers for normal neural stem cells. (**C**) GBM neurospheres exhibit the ability to differentiate.

We cultured and expanded short-term tumor neurospheres in serum-free stem cell media, (supplemented with EGF and FGF), that were derived from four primary GBM patients, (i.e., PBT003, PBT008, PBT022 and PBT030). These GBM neurospheres exhibited a similar morphology to that of normal neural stem cells (Fig. 1A). It is reported that GBM stem-like cells (GBMSCs) and normal neural stem cells (NSCs) share the expression of several markers, such as CD133 and nestin [28]. CD133 is a cell surface marker expressed on normal human NSCs, and its expression is down-regulated in differentiated cell lines. Nestin is an intermediate filament protein produced during development in stem cells originating in the mammalian central nervous system. Co-expression of CD133 and nestin is associated with a poor prognosis for malignant glioma patients [28]. We observed by Western blot analysis that CD133 and nestin were expressed in GBM neurospheres cultured from each of the four GBM patients, while no expression of CD133 and nestin was observed in an established GMB cell line, (i.e., U87 cells), (Fig. 1B). β-actin expression levels were monitored to ensure that equivalent amounts of cell protein were loaded into each lane of the gel. When cultured in media with serum, the neurospheres derived from PBT003, PBT008, PBT022 and PBT030 GBM patients have the ability to differentiate into adherent and neuron-like cells, (Fig. 1C). Cell cultures derived from the specimens of the four GBM patients (PBT003, PBT008, PBT022 and PBT030) grew in typical neurospheres, and retained the ability for self-renewal and expressed neural stem cell markers, such as CD133 and nestin when cultured in serum-free media. These neurospheres also had the ability to differentiate in serum-containing growth media. In this report, we therefore defined these neurospheres as either GBM stem-like cells or cancer stem-like cells.

### BBMD3 Disrupts the Structure of the Neurospheres and Inhibits the Viability of Cancer Stem-like Cells Cultured from the Four GBM Patients

**Figure 2:**
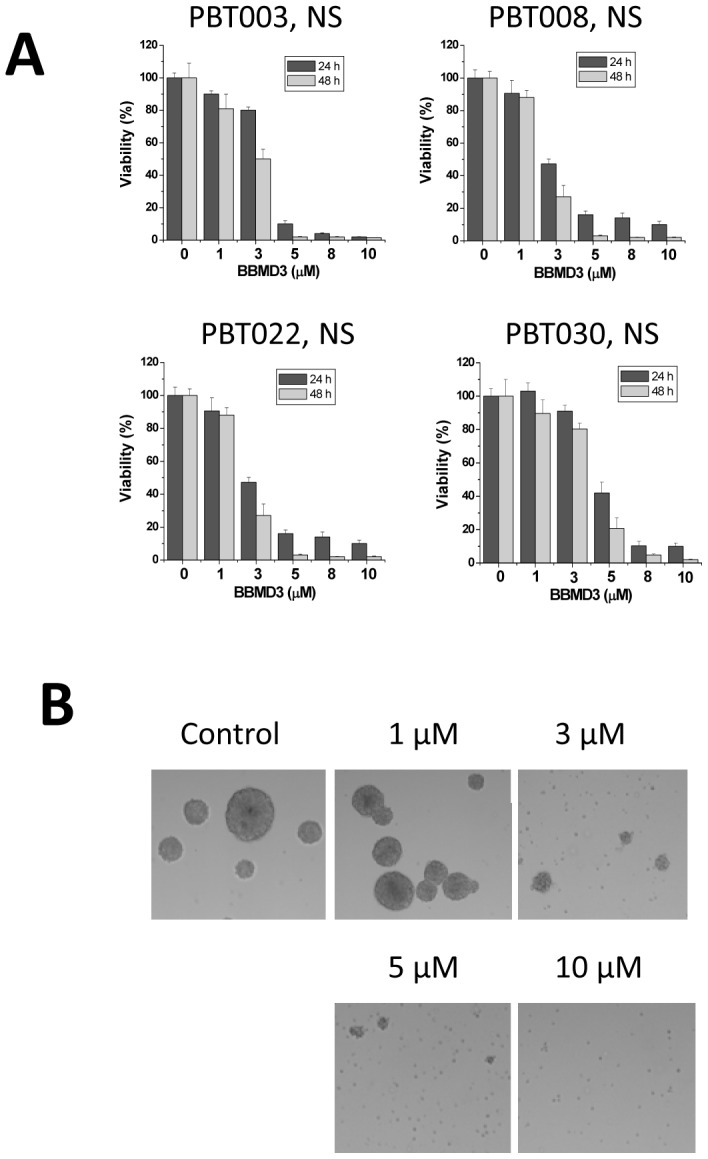
BBMD3 inhibited cell viability and induced cell death in PBT003, PBT008, PBT022 and PBT030 neurospheres. (**A**) Cells from PBT003, PBT008, PBT022 and PBT030 neurospheres were treated with 0, 1, 3, 5, 8, 10 µM BBMD3 for 24 and 48 hours, and viability was determined using the MTS assay, as described in the Methods. Each experiment was performed in triplicate. The top of each bar graph represents the mean of 3 experiments, and the *error bars* represent ± the standard deviation from the mean (SD). (**B**) BBMD3 disrupts the structure of the neurospheres, and induced cell killing in a dose-dependent manner in these PBT003 derived neurospheres, when cells are treated for 48 hours with the drug.

GBM derived cancer stem-like cells, are less sensitive to, (or become resistant to), conventional chemotherapy [4], [5]; highlighting a critical need for finding new and more selectively potent drugs for treating glioblastoma. Berbamine, a natural product derived from the *Berberis amurensis* plant, appears to have cytotoxic activity toward a variety of cancers; prompting the preparation of a series of analogs of Berbamine. These analogs were evaluated to determine whether any of them might be more effective at killing cancer cells than the parent compound. We found that the anticancer efficacy of one of the novel synthetic derivatives, (i.e., BBMD3), on melanoma and prostate cancer cells exceeded that of the other analogs [17]. Thus, we investigated the anticancer effects of BBMD3 on GBM stem-like cells, which were derived from neurospheres cultured from GBM patient’s PBT003, PBT008, PBT022 and PBT030. During the study, these cells were treated for 24 or 48 hours in the presence of complete stem cell culture medium containing 1, 3, 5, 8 or 10 µM BBMD3. Control cell cultures were treated with only vehicle (DMSO) in place of BBMD3, and cell viability was determined as described in the Methods. BBMD3 strongly inhibited the viability of each set of the GBM stem-like cancer cells, derived from the individual patient neurospheres, in a time- and dose-dependent manner (Fig. 2A). A 48 hour treatment of the neurospheres with 10 µM BBMD3 inhibited cell viability nearly 100%, and disrupted the structure of neurospheres in a dose-dependent manner. Figure 2B shows that PBT003 derived neurospheres, treated with 0, 1, 3, 5 or 10 µM BBMD3 for 48 hours, disrupted the spherical morphology of the neurospheres following BBMD3 treatment, and that disruption of the neurospherical shape was companied by the appearance of floating dead cells.

### BBMD3 Induces Caspase-3 Dependent Apoptosis of Cancer Stem-like Cells Cultured from Four GBM Patients

**Figure 3:**
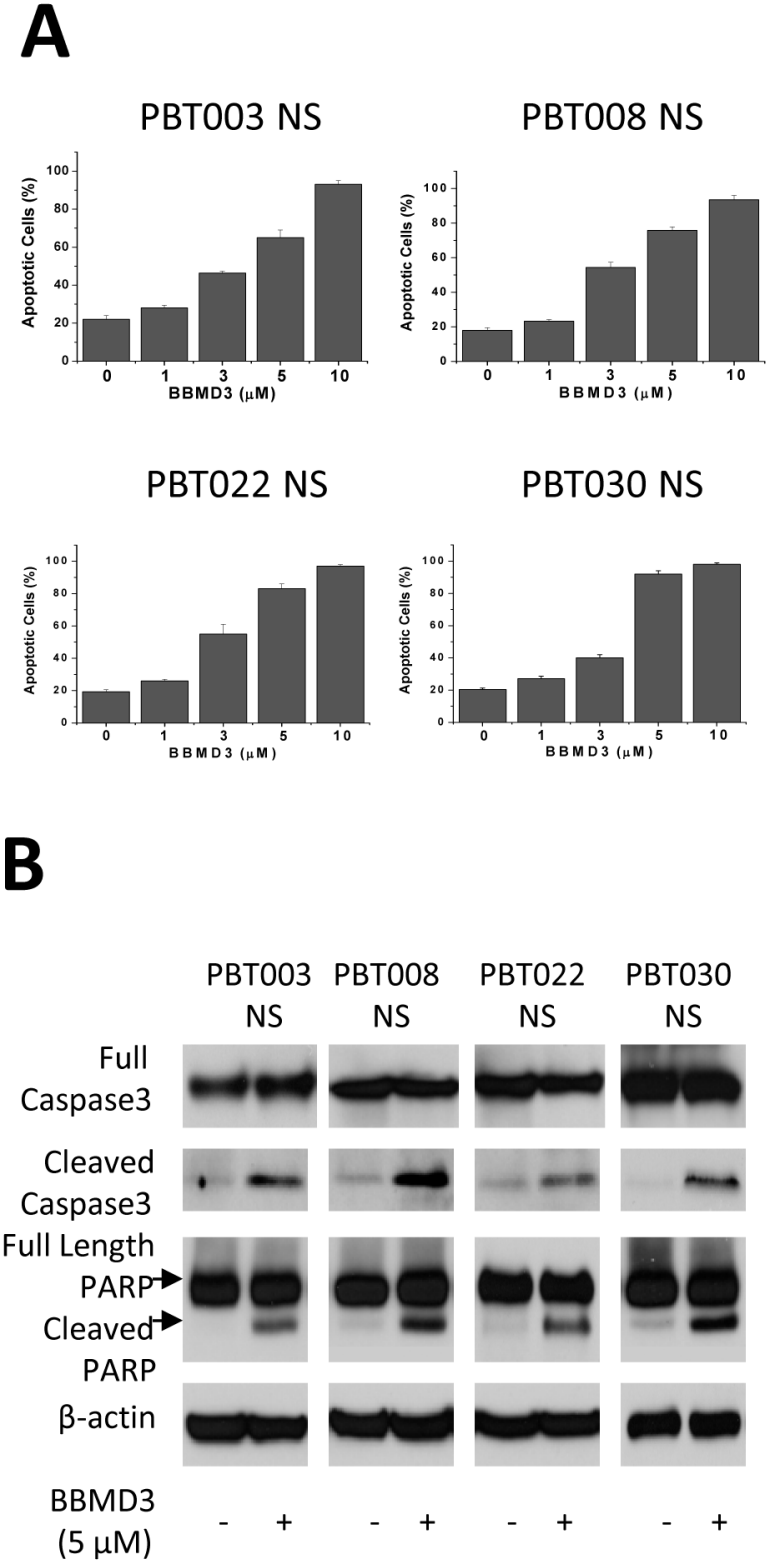
BBMD3 induced apoptosis and increased cleavage of caspase-3 and PARP in GBM neurospheres. (**A**) Apoptotic cells were analyzed for Annexin V-FITC reactivity and PI staining by flow cytometry. Cells from the PBT003, PBT008, PBT022 and PBT030 neurospheres were treated with BBMD3 (0, 1, 3, 5, 10 µM) for 48 hours. Apoptotic cells were identified as being reactive with Annexin V-FITC antibody (early stage of apoptosis) or PI and Annexin V-FITC/PI double-positive (late stage of apoptosis) cells. Each experiment was performed in duplicate or triplicate, and repeated twice as an independent replicate. The top of each bar graph represents the mean of the observed values from these replicates, and the *error bars* represent ± the standard deviation from the mean (SD). (**B**) The extent of cleavage of caspase-3 and PARP was determined following a 24 hour treatment with BBMD3 using Western blotting analyses. β-actin serves as an internal control.

Because BBMD3 treatment killed GBM derived cancer stem-like cells, we examined whether cell killing was mediated by an apoptotic process. Cells (2×10^5^) from PBT003, PBT008, PBT022 and PBT030 neurospheres were seeded into six-well tissue culture plates, and treated for 48 hours with different concentrations (0, 1, 3, 5, 10 µM) of BBMD3. All of the cells were collected and analyzed for expression of Annexin V using anti-Annexin V-FITC antibody and propidium iodide staining followed by relative quantification via flow cytometry. Apoptotic cells, shown in Figure 3A, were Annexin V positive (early apoptotic cells), or Annexin V and propidium iodide double-positive (late apoptotic cells). Our results show that BBMD3 induced apoptosis in these GBM stem-like cells in a dose-dependent manner. A 48 hour treatment with 10 µM BBMD3 killed nearly all of the tumor cells. Activation of caspase-3, a critical inducer of the apoptotic process [29], resulted in cleavage of poly (ADP-ribose) polymerase (PARP), which helps cells maintain their viability [30]. To further confirm that the cell killing induced by BBMD3 is an apoptotic process, Western blotting analyses were performed to detect the activation of caspase-3 and the cleavage of PARP in a total cell lysate following a 24 hour treatment with 5 µM BBMD3. BBMD3 increased the cleavage of caspase-3 into its active form, and the cleavage of PARP into its inactive form in cells derived from the four neurosphere cultures (Fig. 3B). These data point toward BBMD3 inducing apoptosis in these GBM stem-like cells via activation of the caspase-3 cascade. Our data also suggest that multiple mechanisms may be involved in the induction of cell killing and the loss in cell viability, since, at the time point assayed, PBT003 NS incubated with 5 µM BBMD3 induced an almost total loss in cell viability, with an apoptosis rate of approximately 60%; while for the PBT030 NS cell viability was approximately 20% after incubation with 5 µM BBMD3, while apoptosis was almost 100% in this culture.

### BBMD3 also Reduces the Viability and Induces Apoptosis in Non-stem-like GBM Cells

**Figure 4:**
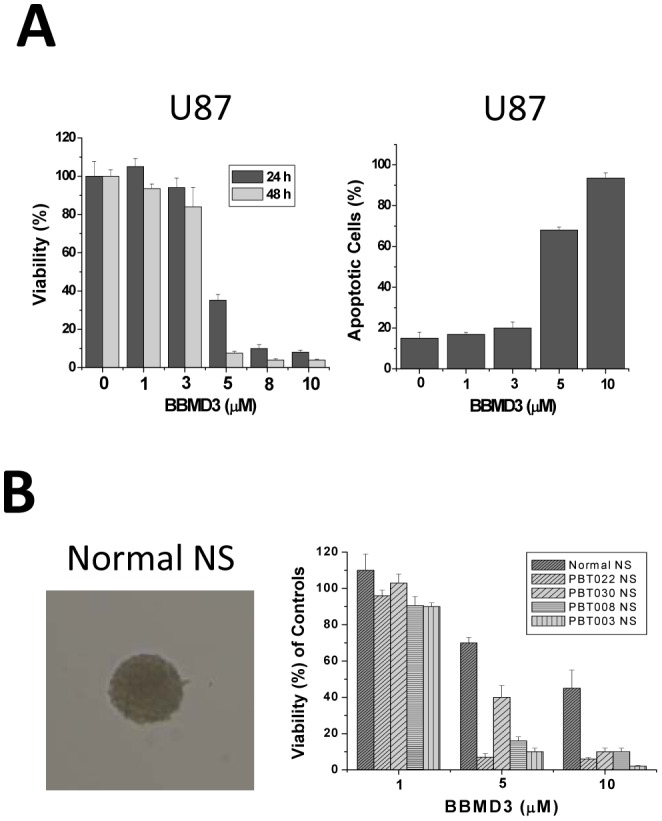
The effects of BBMD3 on non-stem-like GBM tumor cells and normal neurospheres. (**A**) ***Left panel***, U87 cells were treated with 0, 1, 3, 5, 8, or 10 µM BBMD3 for 24 and 48 hours, and viability was determined using the MTS assay, as described in the Methods. ***Right panel***, U87 cells were treated with 0, 1, 3, 5, or 10 µM BBMD3 for 48 hours and apoptotic cells were analyzed for Annexin V-FITC reactivity and PI staining by flow cytometry. (**B**) ***Left panel*** shows the morphology of a normal human neurosphere. ***Right panel*** shows the viability of normal fetal brain derived and tumor derived neurospheres following 24 hours of treatment with 1, 5, or 10 µM BBMD3. Each experiment was performed in triplicate, and replicated as an independent experiment at least twice. The top of each bar graph represents the mean of the observed values from these replicates, and the *error bars* denote ± the standard deviation from the mean (SD).

As we have reported, BBMD3 is cytotoxic to stem-like GBM tumor cells; suggesting that it should also be cytotoxic to non-stem-like GBM cancer cells. To examine this possibility, we used cultured U87 cells, which are an established non-stem-like GBM cancer cell line. We observed that U87 cells did not express the neural stem-cell markers CD133 and nestin (see Fig. 1B for comparison to the stem-like GBM tumor cell lines). As we expected, BBMD3 inhibited cell viability and induced apoptosis in the U87 cells (Fig. 4A) in a similar manner to that seen with the GBM stem-like cells (Fig. 2A and 3A). Our data indicates that BBMD3 can kill both stem-like and non-stem-like GBM tumor cells.

### Normal Human Neurospheres are Less Sensitive to BBMD3 than GBM Derived Neurospheres

Berbamine and its derivatives have also been reported to exert less of a cytotoxic effect toward normal human cells [17] than toward GBM and other types of tumor cells. To further evaluate the differential cytotoxicity of BBMD3 toward normal neurospheres, we tested the effects of BBMD3 on normal human neurospheres derived from the normal fetal brain. Figure 4B shows the morphology of normal neurospheres, and the effects of BBMD3 on normal neurospheres and the neurospheres derived from the four GBM patients following a 24 hour exposure to the drug. In comparison to the tumor derived neurospheres, normal neurosphere viability is reduced by exposure to increasing concentrations of BBMD3, but these normal neurosphere cultures are less sensitive to the cytotoxic effects of BBMD3 than the neurosphere cultures derived from the 4 GBM patients.

### BBMD3 Increases the Expression of miR-4284 and miR-27a in PBT003, PBT008, PBT022 and PBT030 Neurospheres

Though miRNAs have been known for only a few years, they have been found to play crucial roles in the processes mediating tumorigenesis, angiogenesis, cell invasion, and apoptosis in a variety of tumor types. We therefore examined whether BBMD3 treatment could alter the expression profile of miRNAs in GBM stem-like cells. Neurospheres, derived from PBT003, PBT008, PBT022 and PBT030 GBM patients, were treated with 5 µM BBMD3 for 16 hours, while control cells were treated with only vehicle (DMSO). Total miRNA was isolated from these neurosphere cultures, and expression of each miRNA was quantified. In comparison to the controls, BBMD3 treatment up-regulated expression of some miRNAs, and down-regulated expression of other miRNAs. Table 1 shows the average miRNA expression profile, relative to the untreated control GBM stem-like cells, for each of the four GBM patients following BBMD3 treatment. miR-7 is the most down-regulated miRNA, with a decrease in expression of 1.06 fold. In contrast, the two most up-regulated miRNAs are miR-4284 and miR-27a; increasing 4.48 and 2.25 fold respectively. It was reported that up-regulation of miR-23a, 27a, and 24–2 induces both caspase-dependent and –independent apoptosis in human embryonic kidney cells [31]. To date, we have been unable to find any publication describing the regulatory function(s) of miR-4284.

**Table 1:** Effects of BBMD3 on expression of MiRNAs in PBT003, PBT008, PBT002, and PBT030 neurospheres after 15 µM BBMD3.

Downregulation		Upregulation	
Name of miRNA	Folds	Name of miRNA	Folds
hsa-miR-7	−1.06	hsa-miR-7	4.48
hsa-miR-106b	−0.91	hsa- miR-106b	2.25
hsa-miR-25	−0.79	hsa-miR-25	1.26
hsa-miR-20b	−0.77	hsa-miR-20b	1.15
hsa-miR-20a	−0.77	hsa-miR-20a	1.00
hsa-miR-185	−0.64	hsa-miR-185	0.78
hsa-miR-374b	−0.58	hsa-miR-374b	0.71
hsa-miR-503	−0.57	hsa-miR-503	0.70
hsa-miR-148a	−0.56	hsa-miR-148a	0.57

### miR-4284 Inhibitor Blocks the Effects of BBMD3 on the Viability of GBM Neurospheres

**Figure 5:**
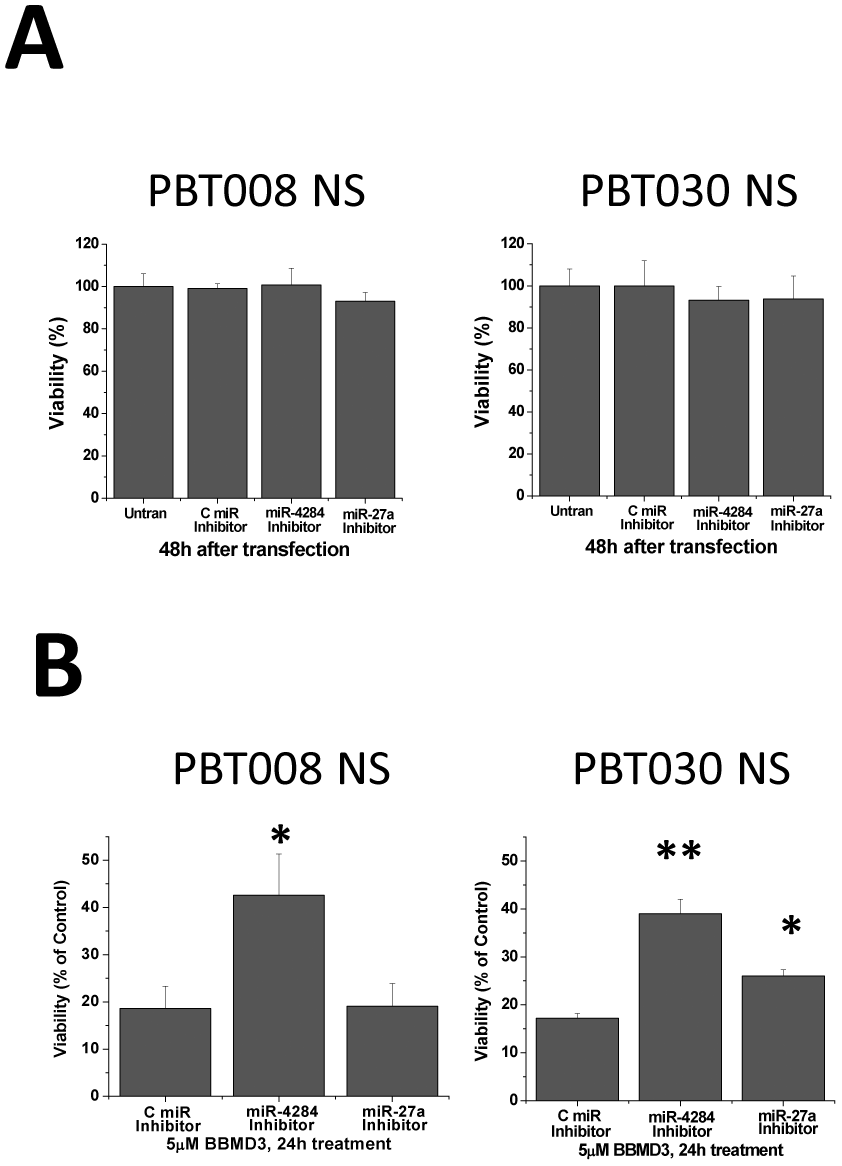
The effect of miR-4284 and miR27a inhibitors on the cytotoxic activity of BBMD3 in PBT003 and PBT030 neurospheres. (**A**) Cells from PBT003 and PBT030 neurospheres were transfected with 60 nM of anti-sense inhibitors against miR-4284, miR-27a and an unrelated control miRNA, and the inhibitor was allowed to interact with the miRNA for 48 hours prior to determining the viability of the cells. (**B**) After cells from PBT003 and PBT030 neurospheres were transfected with 60 nM anti-sense nucleotide inhibitors against miR-4284, miR-27a and an unrelated control anti-sense miRNA, and the inhibitors were allowed to interact with the miRNA for 24 hours; 5 µM of BBMD3 was added to the culture media for another 24 hour. Cell viability was determined at the end of the 48 hour period. Each experiment was performed in triplicate. The top of each bar graph represents the mean of 3 experiments, and the *error bars* represent ± the standard deviation from the mean SD. *, p<0.01; **, p<0.001.

To confirm whether the increased expression of miR-27a and miR-4284 induced by BBMD3 treatment correlates with the decrease in GBM neurosphere viability, we examined whether miR-27a and miR-4284 anti-sense sequences could inhibit the cytotoxic activity of BBMD3. Our first step was to examine whether these inhibitors alone inhibited cell viability. 60 nM of human anti-sense miRNA inhibitors (i.e., synthetic oligonucleotides) against hsa-miRNA-4284 and hsa-miRNA-27a were transfected into cells derived from PBT008 and PBT030 neurospheres. Unrelated anti-sense micro-RNA inhibitor was used as a negative control, and 48 hours following transfection with these miRNA inhibitors, cell viability was determined. The results shown in Fig. 5A demonstrated that miR-27a and miR-4284 inhibitors alone did not effectively decrease cell viability relative to that of the untransfected cells (untran). We then tested the effect of miR-27a and miR-4284 inhibitors on the cytotoxic activity of BBMD3 using PBT008 and PBT030 neurospheres. After these GBM neurospheres were transfected with, and allowed to express for 24 hours, the miR-27a and miR-4284 anti-sense inhibitors, or the control miRNA inhibitor sequence, cells were incubated with 5 µM BBMD3 for another 24 hours, at which point cell viability was determined. The results shown in Figure 5B indicated that, in contrast to the control miRNA inhibitor, cells transfected with the anti-sense miR-4284 inhibitor showed greater viability (two fold) following BBMD3 treatment in both the PBT008 and PBT030 neurosphere cultures. However, transfection of the miR-27a anti-sense inhibitor into these neurosphere cultures was not as effective at reducing the cytotoxic effect of BBMD3 treatment on the GBM stem-like cells as transfection with the miR4284 inhibitor. Therefore, BBMD3 mediated inhibition of GBM stem-like cell viability depends, at least in part, on increasing expression of miR-4284.

### BBMD3 Increases the Phosphorylation of JNK1 and JNK2 in GBM Stem-like Cells

**Figure 6:**
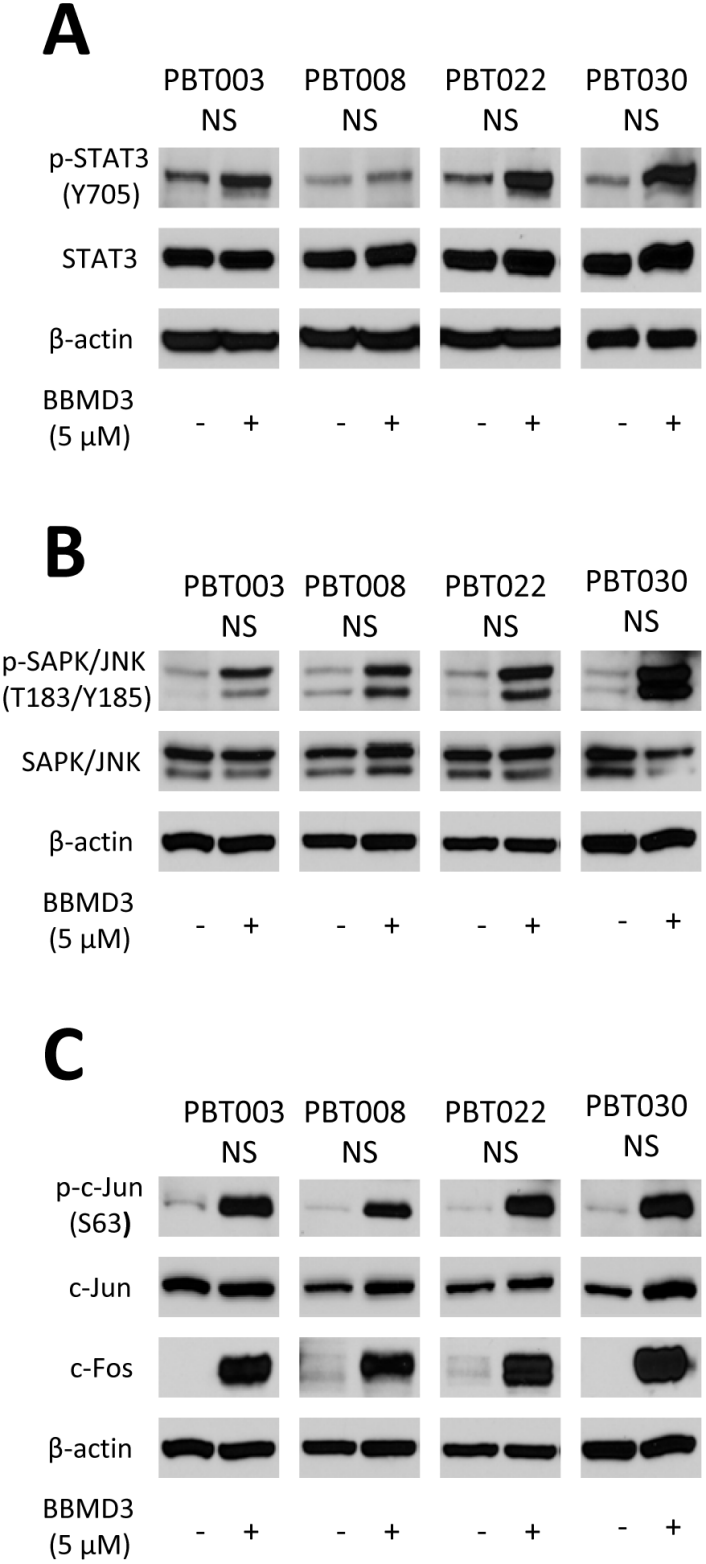
Western blotting analysis of BBMD3 induced phosphorylation of JNK 1 and 2, c-Jun and total c-Fos protein expression in cells derived from human GBM neurospheres. (**A**) The effect of BBMD3 on the expression of total and phosphorylated STAT3 in cells derived from PBT003, PBT008, PBT022 and PBT030 neurospheres following a 24 hour treatment with BBMD3. (**B**) Western Blotting analysis of cell lysates probing for phosphorylated JNK expression following a 24 hour treatment of cells derived from GBM neurospheres with BBMD3. (**C**) Phosphorylation of c-Jun and quantification of c-Fos total protein expression following BBMD3 treatment.

Since BBMD3 was reported to inhibit the STAT3 signaling pathway in melanoma cells [17], we evaluated whether BBMD3 could also inhibit this pathway in human GBM stem-like cells. Following the treatment of PBT003, PBT008, PBT022, PBT030 derived neurospheres with 5 µM BBMD3 for 24 hours, total cellular protein was isolated and analyzed by Western blotting for phosphorylation of STAT3. Our results indicated that BBMD3 did not inhibit the phosphorylation of STAT3 at Tyr705, the active form of STAT3 in these cells (Fig. 6A). Thus, the anti-cancer potential of BBMD3 on GBM stem-like cells was not a result of inhibiting the STAT3 signaling pathway. We then examined whether BBMD3 could activate the JNK stress-response signaling pathway following BBMD3 treatment. In related work from this laboratory, the JNK pathway was shown to be responsible for the anti-cancer effect of BBMD3 in human osteosarcoma cells [18]. Following a 24 hour incubation of cells derived from PBT003, PBT008, PBT022 and PBT030 neurospheres with 5 µM BBMD3, total protein was isolated and analyzed by Western blotting for expression of the JNK1 (46 kD) and JNK2 (54 kD) isoforms, using antibodies that are specific to these proteins. Surprisingly, BBMD3 dramatically increased the phosphorylation (active form) of both of the JNK isoforms in the neurosphere cultures derived from all four GBM patients (Fig. 6B); suggesting that the up-regulation of the JNK signaling pathway mediates the cytotoxic response exhibited by the neurosphere derived cells following incubation with BBMD3.

### BBMD3 Increases the Phosphorylation of c-Jun in GBM Stem-like Cells

Because up-regulation of the JNK/AP-1 signaling pathway was shown to have a direct role in the induction of apoptosis induced by BBMD3 in human osteosarcoma cells [18], this signaling pathway was anticipated to play an important role in the apoptotic process induced by BBMD3 in GBM stem-like cells. c-Jun is a downstream target of the JNK signaling pathway, and is the most extensively studied protein of the activator protein-1 (AP-1) complex. It has an important role in contributing to the regulation of numerous cellular processes, such as cell proliferation, apoptosis, cell survival, tumorigenesis and tissue morphogenesis. Because our data indicates that BBMD3 activates both JNK1 and JNK2, we evaluated whether BBMD3 also activates c-Jun. As expected, BBMD3 contributes to the increased phosphorylation of c-Jun in the neurospheres derived from all four GBM patients (Fig. 6C). BBMD3 also contributed to the increase expression of total c-Fos (Fig. 6C), another major component of transcription factor AP-1.

## Discussion

Glioblastoma is incurable by conventional approaches. Though most of the tumor can be removed by surgery, radiotherapy and chemotherapy, recurrence of the tumor eventually occurs. GBM stem cells play a key role in the initiation of the primary tumors and the recurrence of the tumor following therapeutic intervention. Recurrence is attributable to the self-renewal capacity of the GBM stem-like cells and their inherent resistance to traditional therapy [32]. Thus there is an urgent need for new therapeutic approaches which can selectively target the GBM stem cell. In this study, BBMD3, a novel synthetic derivative from the natural product Berbamine, was shown to effectively inhibit the viability and induce apoptosis in GBM stem-like cells. The anti-cancer effects of BBMD3 in these cells was shown to be associated with the up-regulation of miR-4284 expression and activation of the stress-response JNK signaling pathway, as well as the AP-1 transcriptional factor, a JNK downstream target.

Additionally, microRNAs play crucial roles in the processes mediating tumorigenesis, angiogenesis, invasion and apoptosis for various types of tumors. Recent studies have identified dys-regulation of specific miRNAs in malignant gliomas [10], [11]. In our study, we found that BBMD3 treatment up-regulated the expression of miR-4284 more than four-fold in GBM stem-like cells. An anti-sense inhibitor of miR-4284 activity could partially block the anticancer effects of BBMD3 on those GBM stem-like cells. Thus, BBMD3 inhibits cell viability and induces apoptosis in these stem-like cells, at least in part, through increasing expression of miR-4284. As an aside, it should be noted that all of the functions attributed to miR-4284 expression have not yet been identified. Though miRNA mediated regulation of gene expression remains an emerging field of investigation, expression profiles for a variety of miRNAs have been shown to be useful for predicting GBM patient survival, and these profiles have the potential to identify efficacious therapeutic targets [33].

In this manuscript we reported that BBMD3 reduces GBM stem-like cell viability and induces apoptosis in these cells, in a manner consistent with the reports on the effects of BBMD3 on human melanoma and osteosarcoma tumor cells [17], [18]. These cytotoxic effects of BBMD3 are mediated through inhibition of the Jak2/STAT3 signaling pathway in melanoma cells and activation of the stress-response JNK pathway in osteosarcoma cells, respectively. These reported observations lead to the conclusion that BBMD3 may target different signaling pathways in different tumor types. In our present study, BBMD3 inhibited cell viability and induced apoptosis of human GBM stem-like cells with an LD_50_ of less than 5 µM after a 48 hour exposure. However, BBMD3 did not suppress STAT3 activity, but rather strongly activated the JNK signaling pathway in GBM stem-like cells. The JNK signaling pathway contributes to cell proliferation and the apoptotic process; suggesting that the JNK pathway is involve in the induction of apoptosis in the GBM stem-like cells. The involvement of the JNK pathway during the induction of apoptosis comes from the observation that *Jnk1^−/−^Jnk2^−/−^* mice were resistant to apoptosis induction following UV irradiation, exposure to anisomycin or the DNA-alkylating agent methyl methanesulfate [34]. JNK is an intrinsic component of the mitochondrial-dependent death pathway associated with stress-induced apoptosis. Following activation of JNK, BBMD3 increases expression of phosporylated c-Jun protein and the expression of c-Fos. Both of these proteins are downstream targets of the JNK pathway in GBM stem-like cells. c-Jun and c-Fos are themselves major components of AP-1, a transcription factor, which regulates proliferation, inflammation, differentiation, apoptosis and cellular migration processes [24], [25]. Our data concerning the GBM stem-like cells along with additional evidence [35] indicates that the JNK-c-Jun/AP-1 signaling pathway plays a key role in mediating the induction of apoptosis in GBM stem-like cells [35].

The GBM stem-like cells we used for this project were cultured from four GBM patients and though derived from tumor tissue, retained some of the properties of normal neural stem cells, such as their spherical morphology, the ability to exhibit self-renewal and the ability to differentiate. BBMD3 not only induced apoptosis in these GBM stem-like cells, it also induced apoptosis in established GBM cancer cell lines, including U87 and U251 cells (data not shown). Our data indicates that BBMD3 exhibits a strong anticancer cell activity toward GBM stem-like cells; while exhibiting a lower cytotoxicity toward normal human cells [17]. We therefore believe that BBMD3 may have the potential to be developed as a promising drug for the treatment of malignant gliomas.
