# Correction: A Novel Berbamine Derivative Inhibits Cell Viability and Induces Apoptosis in Cancer Stem-Like Cells of Human Glioblastoma, via Up-Regulation of miRNA-4284 and JNK/AP-1 Signaling

**DOI:** 10.1371/journal.pone.0100708

**Published:** 2014-06-18

**Authors:** The PLOS ONE Staff

Dr. Yuelong Ma is not included in the author byline. She should be listed as the sixth author and affiliated with Molecular Medicine, Beckman Research Institute, City of Hope Comprehensive Cancer Center, Duarte, CA. The contributions of this author are as follows: Contributed reagents/materials/analysis/tools.

Dr. Jun Xie is not included in the author byline. He should be listed as the seventh author and affiliated with Molecular Medicine, Beckman Research Institute, City of Hope Comprehensive Cancer Center, Duarte, CA. The contributions of this author are as follows: Contributed reagents/materials/analysis/tools.
